# Sentience, Harmony and the Value of Nature

**DOI:** 10.3390/ani13010038

**Published:** 2022-12-22

**Authors:** James William Yeates

**Affiliations:** World Federation of Animals, Boston, MA 02130, USA; james.yeates@wfa.org

**Keywords:** biodiversity, harmony with nature, sentience, United Nations, value of nature

## Abstract

**Simple Summary:**

Many people are concerned both about harms to nature and about the suffering of animals. These two concerns are closely related, even if the the links are not aways fully recognised, for example, within UN frameworks. Animals’ feelings are part of nature and part of what makes nature valuable, diverse and healthy. They also represent how animals evalute nature themselves. They can be recognised and assessed to improve our scientific assessment of environmental impacts, decisions and policies, in ways that go beyond human financial interests and to motivate greater collaboration and transformative action to protect nature.

**Abstract:**

Concern for nature and for animal sentience are important public and political moral concerns. Using frameworks such as Harmony for Nature and One Health and the recent IPBES report on the Diverse Values of Nature, this paper considers how the two issues interrelate, in terms of our concepts of sentience and nature, and sentience-based values’ importance in relation to nature-based values. Animals’ sentience is part of nature, and part of its diversity, harmony, health and value. Sentient animals’ feelings represent animals’ evaluations of nature that go beyond valuing nature for solely for market-based and anthropocentric interests. Sentience is therefore relevant for measurement, leveraging and embedding sentience-based values in environmental concerns, including in environmental impact assessments, science-based UN policy-making, interdisciplinary and interagency collaboration, and to strengthen transformative and system-based action for nature.

## 1. Introduction

Concern for nature and for animal sentience is important ethically and politically, at individual, national and international levels. For example, the UN General Assembly has recognised the value of both in the series of resolutions on Harmony with Nature which, since 2009, has increasingly defined a position that includes protection of the ecosystems, species, biodiversity and sentient animals. In the recent 2020 Report, the UN Secretary-General stated:

“A first step to recognizing the rights of Nature is the recognition that non-human animals are sentient beings, not mere property, and must be afforded respect and legal recognition. Such recognition is growing around the world, in particular with regard to those animals best known and most easily appreciated by humans” ([1], art. 42).

Recently, the Food and Agriculture Organisation of the UN, the World Health Organization, the World Organisation for Animal Health and the UN Environment Programme recognised the importance of One Health [2], approving a definition that includes the health and wellbeing of animals [3], which can be taken to include mental and social wellbeing [4], and which was recognised by the Ministerial Statement of the 2022 UN High Level Political Forum [5]. The UN Environment Programme has also recognised the links between animal welfare and health in a landmark report [6] and animal welfare, environment and sustainable development in a landmark Resolution at the UN Environment Assembly in 2022 [7].

It is important to ascertain how sentience is relevant to our concern for nature because protecting nature is a core focus of international policymaking, which needs to be transformational if it is to adequately protect nature, ourselves, future generations, and animals who share our planet. It is therefore important to identify all concerns that may help strengthen action, and to recognise the importance of each in their own right and collectively. However, while there has been some consideration of the links between sentience-related values and natural behaviour, e.g., [8] or naturalness, e.g., [9] there has been relatively little consideration of how recognising sentience is relevant for valuing nature.

This paper considers the potential significance of animal sentience in our relationships with nature. The first part considers sentience as part of nature and of prevalent narratives and worldviews concerning nature. The second part considers the importance of sentience in relation to recognising the values of nature, measuring value, leveraging those values in transformation, and embedding them in decision-making. It draws on concepts from the agendas on Harmony with Nature, One Health, and Biological Diversity, including the recent Intergovernmental Platform on Biodiversity and Ecosystems Services (IPBES) report on the Diverse Values of Nature [10], which was written to comprehensively identify values beyond predominant instrumental values of nature that are consistent with living in harmony with nature, but which omitted any consideration of values related to animal sentience. The paper’s conclusions are therefore informative for the achievement of those agendas, and thus to the strategies of the UN General Assembly, the UN Environment Programme, the Convention on Biological Diversity, the Food and Agriculture Organisation, the World Organisation for Animal Health, the World Health Organisation and the One Health High Level Expert Panel.

## 2. Understanding Sentience as Part of Nature

### 2.1. Sentience as Part of Nature

Sentience is a capacity to have feelings [11] and specifically those that matter to each animal [12]. These are, in particular, motivational and affective states, which may be negative or positive, including preferences, aversions, frustration, fear, pain, affection and enjoyment. Sentience is a natural capacity that has emerged (perhaps multiple times) through evolution. The presence of animal sentience, and of specific animal feelings, are facts about the world (which we may or may not recognise). Animal sentience is therefore an aspect or part of nature: specifically, it is a capacity of components of nature, like flying and metamorphosis. Animals’ feelings themselves are also parts of nature, as are the resources, rewards, threats, conspecifics and other factors that impinge on an animal’s feelings, and the parts of nature that are affected by sentient animals’ responses.

Animals’ feelings are embedded within natural processes insofar as they affect how animals interact with the world. Animals’ affective states colour their perception and understanding of the world (or “their world” as they perceive it). Animals’ affective and motivational states drive their behaviours including mate selection, food choices and displacement decisions, as animals make trade-offs, exhibit goal-oriented flexibility, and learn through operant conditioning [13]. Motivated behaviours are relevant ecologically as factors for animals’ survival, their impacts on their surrounding ecosystems, and their contributions to wider processes such as carbon, nitrogen and water cycles. Such motivations are also determinant of how ecosystems naturally change (alongside changes due to insentient processes), as species migrate, alter their behaviour and, over time, and evolve through their survival and mate selection.

Sentience is a relational capacity, linking animals to their environments. Animals live from, with, in and as nature (after [10]). Animals’ feelings appear to have causal relationships with their environments. In one direction, they are mediated by animals’ perceptions of their environments that are at least partly affected by the reality of those environments. In the other direction, these relationships are mediated by animals’ motivated operant behavioural responses (which have effects on those environments). Sentience may also be of particular relevance in relationships between sentient animals as intersubjective connections between individuals, mediated by feelings such as affection and fear that manifest themselves in reproductive, parental, agonistic or antagonistic behaviour. Some sentient animals may also have the capacity for empathy in response to the perceived feelings of other animals, linking sentient animals to others.

Sentience also constitutes a significant addition to the complexity of nature. It is an emergent property that is more than the sum of each animal’s neuro-endocrine or biochemical parts. Sentience brings about a new level of phenomenon that is within nature, but which is different in kind to many other natural phenomena.

### 2.2. Sentience as Part of Key Attributes of Nature

Animal sentience also contributes to the diversity of nature. As an additional property to the physical elements of the environment, the sentience of some organisms adds an additional dimension and degree of variation between and within species. Sentience means nature is diverse beyond species’ genetics, insofar as sentient individuals each have different subjective experiences or viewpoints. Nature is therefore diverse in part because an animal’s feelings are part of nature, and animals will each have different feelings, different emotional predispositions, and diverse capacities to have feelings. Because of this, sentience can sometimes help to describe or explain intraspecific variation where individuals choose differing tactical responses, e.g., [14].

Sentience is also an aspect of the integrity and harmony of nature. Alongside considering the integrity and harmony of nature in taxonomic terms (e.g., the balance of species within an ecosystem), we might also think of sentient animals’ feelings being an integrated and overall whole, and in dynamic equilibrium over time. No sentient animal can (probably) be without suffering, but their overall experiences may constitute a more or less balanced whole, without any one type of experience disproportionately dominating or excluding others. The UN idea of harmony with nature also reflects our relationships with the environment and animals, and having harmonious relationships *with* nature might be taken to include ensuring harmony *within* nature and not causing mental health imbalances in sentient animals. Similar considerations might apply to concepts such as “making peace with nature”, in which UNEP recognise the need to improve animals’ welfare [15].

We might further describe animal sentience as an aspect of the health and needs of the environment. Under a functional perspective on health, animals’ capacity for feelings might be seemingly removed (e.g., coma) or altered (e.g., knock-out pain-free animals). Sentient animals’ feelings are also, of course, affected by poor health from symptoms such as malaise and pain due to disease or starvation. Sentient animals may also experience mental health issues such as depression and anxiety, which are recognised in domestic and captive animals but relatively unstudied in free-living wild animals. Similarly, sentience affects what animals need in that it engenders not only physical needs for survival and growth, but also mental needs to avoid suffering. A decrease in mental health or an increase in negative feelings of suffering may therefore be seen as *indicators* or *predictors* of environmental damage.

### 2.3. The Role of Sentience in Human-Nature Interactions

Animal sentience is an important part of how environments are affected by humans, and how they respond. Firstly, the feelings of sentient animals, in particular affective states such as suffering, as responses to their environments, are potential symptoms of the effect of human actions and anthropogenic changes. Causing disproportionate suffering in sentient animals is an indicator that our relationship with nature is far from harmonious. Secondly, animals’ motivations affect animals’ behavioural responses to human impacts such as human presence or environmental disruption, which in turn can affect their health, flourishing or survival as well as affect how human–animal interactions play out. These welfare effects can, in turn affect human health [6]. More positively, sentience is part of nature’s forces that maintain itself, and sentient animals’ psychological resilience is part of the environment’s overall resilience. Nature is therefore not a neutral and passive “blank canvas” for human action, but one which actively and intentionally responds to human interventions (unless prevented from doing so by other human interventions).

Sentience also bridges humans to nature. Sentience allows us to recognise similarities to our nature within nature more widely, particularly when we see ourselves as living ‘in’ and ‘as’ nature (following [10]). Animals are similar to humans as regards being sentient (in a way that we believe we are dissimilar to natural phenomena such as rocks and meteorological events). Our shared sentience appears to have pre-evolved our differentiation into *Homo sapiens* and other sentient species, and is possibly also the result of convergent evolution across multiple taxa [16]. This suggests that our shared sentience is phylogenetically prior to—and independent of—our higher cognitive abilities. It also means that we, as sentient animals, have remained natural in our sentience, however much cultural and technical change we have since undergone. The recognition of animals’ sentience therefore provides a way to bridge the perceived distinction between “humans” and “nature”. It makes part of nature less different to us.

The consideration of sentience similarly reminds us that domestic animals are, in a very important way, natural. Insentient substances might, perhaps, be said to be taken out of nature and rendered human artefacts (e.g., plant fibre for paper). Animals, as sentient beings, retain at least this natural property insofar as their sentience is one aspect of them that is not changed by human interventions. Whether animals are in human captivity, undergo bodily mutilations or have aspects of their phenotype and genotype selected by humans, they all still constitute part of nature. More specifically, many captive and domestic animal are able to experience the same types of feelings (pain, pleasure, etc.) and motivations (aversion, curiosity, etc.) in similar circumstances (injury, threat, confinement etc.). What changes is the actual feelings that they experience as a result of anthropogenic impacts. Part of the impact of human activity on nature is the suffering and other feelings that sentient animals experience as a result.

## 3. Understanding the Sentience-Related Diverse Values of Nature

### 3.1. The Value of Sentience and Nature

Animal sentience is one way for us, as humans, to connect with nature—a way which has probably developed over millennia and in humans around the world. Sentience-related values may include both a broad concern for sentience in general (e.g., a widespread compassion), and specific concerns (e.g., the pain experienced by animals caught in snares or the frustration of animals in close confinement). These values may be expressed on their own, or in combination with concerns for other humans’ needs or nature as a whole. Indeed, people may not rigidly differentiate or select between, say, animal and environmental protection, but see them as complementary and interconnected aspects of a broad value.

How then might sentience have value? Firstly, sentient animals are valuable and worth protecting as a part of nature. However, we might go further. Sentience is a special part of nature, with which only some parts of nature are endowed, and deserving of particular attention. Sentience also means that parts of nature are irreplaceable insofar as each sentient animal is an individual with a unique set of experiences in addition to their nature as a member of their species (which philosophers might describe as their haecceity and quiddity, respectively). Furthermore, the fact that animals have their own experiences makes nature even more of a wonder and mystery, enhancing the numinous awe for those able to access it, in ways that go well beyond instrumental market values but capture the appreciation of, say, those who enjoy watching wildlife engaging with their natural surroundings.

Secondly, sentience means animals have feelings which have value. Feelings may be seen as having intrinsic value, insofar as (for example) pain is inherently bad and its absence to be innately desired [17,18], and motivations are per se (albeit *ceteris paribus*) worth fulfilling. Since these feelings are part of nature, this is one way in which nature has intrinsic value. Since these feelings are engendered by nature, this is one way in which nature has instrumental value for those animals in it (or deprived of it) as well as for ourselves. So, whether we see animals as living ‘from’, ‘with’, ‘in’ or ‘as’ nature, ecosystems make contributions to animals too, including the functions, structure, and ecosystem processes that affect their feelings and responses.

Sentience also means nature has valuers. Sentient animals value their own states (e.g., their health) and their environments (e.g., resources and threats). They show their valuing (or at least their evaluations) by physiological reactions such as stress-responses, behaviours such as vocalisations, tongue protrusion, facial expressions, posture, limb withdrawal, as well as intentional approach, avoidance, resistance, and choices to consume or interact with aspects of their environment. This recognition of valuers within nature provides one way to avoid the challenge that, if we think value needs a valuer, then nature cannot have value unless humans value it (as played out in fantastic thought experiments about what would be morally acceptable for the last human on earth).

This consideration of sentience affords nature both positive and negative value, depending on animals’ overall experiences, in particular the extent and severity of suffering. As humans alter nature in ways that lead to animal suffering, this reduces the value of nature (but not the potential value of nature if we reverse those changes). Indeed, the recognition that nature has value for animals is a way to move beyond anthropocentric concepts of instrumental value for humans to consider more other-relative (and thus less self-oriented or even selfish) values. A good example is the concept of One Health cited by the IPBES Values report (Box SPM.7), which has recently been expanded to include explicit consideration of animal, plant and environmental health and wellbeing by the One Health High Level Expert Panel and the quadripartite of the Food and Agriculture Organisation, the World Health Organization, the World Organisation for Animal Health and the UN Environment Programme, with animal wellbeing closely related to animals’ sentience [3].

The feelings of sentient animals may be related to trade-offs (e.g., by animals themselves in elasticity of preference studies), aggregated (e.g., in welfare at farm and group level assessments) or compared (e.g., in cost–benefit analysis in animal experiments project licence applications). Indeed, considering feelings may provide a way for human and animal interests to be considered together, in that the underlying phenomena are theoretically comparable or compatible. However, global economics have generally considered only the narrow instrumental value of animals, particularly as products, and ignored the externalities associated with the negative impacts on sentient animals. Nonetheless, many human norms, social conventions, laws and institutions are underpinned by sentience-related values (e.g., animal cruelty laws and civil society campaigns), which shows society does consider that sentience-based values should not be subordinated to commercial or other human interest.

### 3.2. Measuring and Making Visible Sentience-Based Values of Nature

If nature has value in relation to sentient animals, then this value is *per se* logically independent of whether we respect it. Nonetheless, the ascription of sentience to animals may help us to recognise value in nature, in particular to improve our ability to assess value beyond our anthropocentric viewpoints and methods of evaluation.

The feelings that human activities engender in animals (domestic and wild) are themselves aspects of their environmental impacts. Assessing suffering provides useful data on the severity and extent of anthropogenic impacts (i.e., in addition to mortality and other data), and may identify suffering that “coarser” population-level environmental impacts miss. By the same token, effects on animals’ health-related feelings and mental health may be seen as part of the health of their environment, and their psychological balance as part of the harmony of the environment. If a human action causes many or more animals to suffer (disproportionately to the amount of enjoyment), this suggests a negative impact of that action, worse environmental health or greater disharmony. The evaluation of impacts on sentient animals (i.e., animal welfare impacts) is therefore potentially valuable data sets for the assessment of environmental impacts.

In addition, environmental impact assessments may draw on animals’ own evaluations, as evinced by their feelings. Sentience may be seen as a functional capacity that assists animals to assess their environment themselves. Affective and motivational feelings constitute or represent animals’ evaluations of their state and the state of their environment, including their available resources, threats, conspecifics and health, and any anthropogenic effects thereon. Animals demonstrate those evaluations in their behaviours such as avoidance, approach and operant responses.

We can look to sentient animals’ feelings to inform our evaluations. Feelings may be seen as (for us) indicative of whether an impact is good or bad. They may be considered valid and relevant evaluations as coming from the insider point of view of indigenous stakeholders embedded within the context of their environment, and whose emotional evaluations are ecologically determined, ontogenically developed and evolutionarily shaped, and who are arguably best placed to make many evaluations [19]. Each feeling is only a single stakeholder view, which provides a *prima facie* indicator, and we might feel increased confidence when many sentient animals appear to feel similarly about an impact or situation, analogous to high interobserver reliability. Inclusiveness to animals’ valuations might also enhance human stakeholders’ perceptions of the legitimacy of the valuation process, in comparison to the human-centred values “imposed” on nature by market or political considerations.

We might draw on sentient animals’ evaluations to help make comparative evaluations of scenarios that go beyond human monetary considerations. Both animal welfare science and behavioural ecology study how animals make trade-offs, assessing the relative strengths of their motivations for different or composite outcomes (e.g., willingness to pay in terms of effort or willingness to undergo adverse stimuli to attain a goal). While many of the motivations studied have seemed relatively inelastic, such studies may help us evaluate which aspects of the environment are more valuable to animals within those environments.

There is now a wide portfolio of established and agreed-upon valuation procedures for assessing animal feelings, built up over the last four decades (as there are for environmental valuation methods), particularly within veterinary, animal welfare and ethological scientific disciplines. These include biophysical measures (e.g., herd health assessments and physiological measures), behavioural measures (e.g., ethograms and stimulus-response tests) and the experience-based methods used by animal keepers to evaluate the experiences of their own animals, often built up over generations of indigenous and hands-on animal keeping. These different methods can provide complementary information that can be usefully considered together, so long as this does not misrepresent compassionate non-scientific views and valuations or decouple them from their appropriate context.

### 3.3. Leveraging the Diverse Values of Nature for Transformative Change

Valuation and policy-related dialogue can draw on animal welfare science, alongside indigenous, community and other civil society views, giving credence to the knowledge and values of compassionate animal carers and smallholders in co-designing initiatives. However, there are (as for environmental concerns) significant power asymmetries between the humans who articulate instrumental values and the less powerful humans and animals who are affected by practices such as deforestation, intensive farming, industrial fishing and commercial wildlife trade. The perspectives of animals and carers can be ignored, marginalised or conceptually and emotionally alienated, which can engender mistrust, resentment, protest or resistance, including from animals themselves. These might be avoided by a recognition of their sentience [20].

The current dependency of political and economic decisions on a narrow set of values of animals underpins both the global animal welfare crisis and the issues of environmental damage, inequality, and food insecurity for humans and animals. Even many environmental protection approaches tend to focus on the instrumental value of nature for humans [10]. Prevalent economic and legal norms currently promote a restricted set of instrumental values associated with short-term economic profits and political gains.

Incorporating sentience-based values and perspectives into policy design and implementation can help address the negative effects of people’s actions on animals. Considering sentience-based values can help find ways to consider a variety of values of nature. It may also help to bring together the fields of environmental protection and animal protection, providing opportunities to identify synergies, solutions and co-benefits, as an aim that has been identified as needed [21,22,23,24]. At the very least, it means animal protection stakeholders should consider environmental impacts, and environmental protection stakeholders should consider the feelings of animals, both wild and domestic.

Sentience provides a way to include animals in human environmental decision-making. Many ethical approaches consider animal sentience, whether aggregative (e.g., hedonic or preference utilitarianism) or individualistic (e.g., sentience-based rights). A harmonious relationship with animals can be conceived as one that allows them to experience a balance of feelings. A sentient life is not in harmony if suffering unnaturally predominates above positive experiences. Nor is it in harmony if it does not facilitate balanced relationships between individuals. The harmony within as well as between each sentient individual is a part of the harmony of the whole. By the same token, the harmony of our relationships with each individual is part of our harmonious relationship with the whole. The consideration of individual sentient animals is not incompatible with the consideration of ecosystems or nature as a whole, any more than considering individual humans is incompatible with considering the health or sustainable development of humanity worldwide.

Similarly, the consideration of sentience provides ways for trade-offs to be made within policies. The focus on suffering helps us to prioritise more basic needs such as diet and health and, as such, to negotiate compromises among human and nonhuman stakeholders’ different interests and values towards achieving fair outcomes, and thereby “optimise” health and wellbeing across human, animal, plant and environmental health. We may think that the need to avoid severe suffering is more important than our needs for, say, short-term financial profit for large corporations.

Reversing our impact on sentient animals—domestic and wild—will require a systemic, transformative change of established legal and economic norms and rules. Transformative change is more likely to occur when institutional change is widely supported by societal goals. Transformative change to address the global biodiversity crisis “is more likely to be catalysed through actions that target a combination of values”, “recognizing the multiple ways in which people relate to each other and to nature” and “mobilizing broad values that are consistent with living in harmony with nature” [10]. This means respecting animal sentience alongside other values, informing decisions by producers, consumers and policymakers and redesigning systems and governance structures.

### 3.4. Embedding Sentience-Based Values of Nature in Decision-Making about Nature

There are various ways in which concern for animal sentience might improve decision making about nature, including decisions about nonwild animals who are still part of nature, albeit under direct human control.

Concern for sentience has already been recognised by major policymakers, and the consideration of animal welfare in international law is growing [25]. The African Union’s animal welfare strategy aims for “treating animals as sentient beings” [26]. The Lisbon Treaty of the European Union affirms that “States shall, since animals are sentient beings, pay full regard to the welfare requirements of animals” ([27], art. 13). The World Organisation of Animal Health (previously the OIE) has a growing series of international standards in animal welfare since 2000 [28]. The United Nations has recognised its importance in Harmony with Nature [1]. The UN Environment Programme has recognised the links between animal welfare, environment and sustainable development [6]. The quadripartite of the Food and Agriculture Organisation, the World Health Organization, the World Organisation for Animal Health and the UN Environment Programme have endorsed a definition of One Health that includes animal, plant and environmental wellbeing. The World Trade Organization has recognised animal protection as a relevant matter of public concern that could justify restrictions on trade [29].

Animal welfare science is an important knowledge system for protecting nature. Sentience, as a natural capacity, can be—and is—investigated by scientific methods. We need to understand animals’ feelings to be able to predict and understand the complexity of nature, our anthropogenic impacts on it and the responses and adaptions of sentient animals. This must be alongside other scientific disciplines (e.g., relating ethology and behavioural ecology) and sources of knowledge (e.g., relating scientific and indigenous knowledge), which need to be brought together in an inter-disciplinary understanding based on nature’s diverse values. However, there are significant knowledge gaps amongst key stakeholders, potentially structurally embedded for example by industry marketing or court rulings that aim to censor whistleblowing about the harms of industrial agriculture [30], which need to be overcome.

Sentience is potentially a powerful approach for mobilising the necessary changes in individual human stakeholders. Sentience evokes an emotional response in most humans, of empathy and compassion. As for sentience itself, these responses may be both affective and motivational. Affectively, sentient animals are the part of nature with which we can genuinely empathise as being, like us, able to suffer. We can (to some degree) understand and share these feelings, while also recognising animals have their own feelings and values that matter beyond our own interests. Motivationally, sentience helps us to generate other-directed and compassionate motivations. 

Many environmental concerns have a significant component of concern for the animals. We are faced with images of elephants displaced into cities, turtles caught in ghost nets, birds entwined in plastic, koalas and lizards burnt by forest fires, frogs suffering from chytrid disease, wildlife caught in snares, and polar bears and marine fish deprived of habitat. Reverting or reforming land use change, pollution, unsustainable wildlife hunting and intensive farming can be motivated by concern for the suffering of animals affected. The prevalent, positive and successful use of charismatic megafauna in environmental campaigning (and branding) is partly because we can relate to such animals—pandas, whales, turtles, leopards, orangutans—as able to suffer, as experiencing their environment and our impacts on it, and whose unique perspectives are an irretrievable loss when destroyed. Highlighting effects on sentient animals may well be a potent way to strengthen action for nature, for example in relation to diet change to reduce GHG emissions, water pollution and deforestation [31].

Different stakeholders act on different values (often also influenced directly or indirectly by power relations and incentives). We might hope to see greater collaboration between societal actors to promote system-wide, values-centred transformations, to achieve the 2030 Agenda for Sustainable Development and implement the post-2020 Global Biodiversity Framework. For example, the recognition of animal sentience highlights the need for IPBES and Convention on Biological Diversity work to align with the Harmony with Nature and One Health agendas and the UN Environment Programme’s strategy to ensure coherence between them. For example, the IPBES current anthropocentric conceptualisation of One Health ([10], Box SPM.7) needs to be re-aligned with the official quadripartite definition cited above that includes reference to animal health and wellbeing. Such collaboration is beginning to be evinced already by some civil society representatives (e.g., the Major groups and Stakeholder groups that feed into the UN), non-governmental organisations (e.g., members of the Species Survival Network, 50by40, and the Pandemics and Animal Welfare Coalition) and nascent inter-agency work such as the One Health High Level Expert Panel. Considering sentience could help to develop and align worldviews and values which adequately reflect sentience alongside other values of nature.

Moving forwards, considering sentience might prompt integrative interventions needed to drive transformative change, such as the elimination of harmful subsidies or tax exemptions to large scale animal exploiters, resource-intense producers, and polluters. We might even, in time, see more integrative or ecological concepts within economics (e.g., to include animals’ behaviourally expressed motivations and connections between individuals, or to concepts of quality of life or prosperity applicable to humans and animals).

Transformative change will also require changes in political approaches and governance. Explicit consideration of sentience-based values might help inspire a shift from self-oriented and short-term political approaches. An anthropocentric approach is, in essence, an exclusive and restrictive approach. As such, it is in the same family as excessive partiality towards, or prioritisation of, each individual’s country, race, family or self. Broadening the recognised scope of our concern and compassion may also help to reinforce, deepen and activate our concern and compassion for all humans as well as animals. It is beyond the scope of this paper (or the IPBES report) to imagine what such an approach might look like, although we can identify a need (as the IPBES report does) to consider a diversity of values, to address power asymmetries, to recognise how the benefits and burdens arising from these decisions are distributed, and to see the potential for sociocultural and rights-based instruments to support systemic transformations that respect all humans, animals and the environment.

## 4. Sentience in International Policy

Concern for sentience is an important part of many global concerns. It is a strong element of harmony with nature, insofar as a harmonious relationship must be one that avoids cruelty and unnecessary suffering and, more positively, a relationship of respect and compassion that considers sentient animals’ needs and mental health and wellbeing. This includes whether those animals are still in nature as wildlife or are part of nature removed into anthropogenic environments as domestic or captive animals.

Similarly, a concern for One Health, contextualised by both the long-standing World Health Organization definition and the more recent quadripartite definition of One Health, needs to consider animals’ mental health, emotional wellbeing, and the impact of animals’ physical health on their feelings. To this end, the United Nations Ministerial Declaration for the High-Level Political Forum in 2022 emphasised the importance of One Health and other holistic approaches that deliver multiple benefits to the health and well-being of people, animals, plants and ecosystems [5]. The quadripartite approach to One Health has generated a Global Plan of Action aimed to improve the health and wellbeing of humans, animals, plants and the environment [32] that should be implemented in ways that afford appropriate protection to sentient animals.

By the same token, a human right to a healthy environment logically includes healthy animals as part of our environment. This means keeping animals at least naturally healthy. It also means ensuring animals’ environments—which they live in, from, as and with—are healthy for them.

The concern for protecting nature, usually focused on ecosystems and biodiversity, also needs to be aligned with sentience-based concerns. The recent IPBES report’s robust and systematic typology of the diverse values of nature is welcome as recognising the importance of considering a comprehensive range of ways in which nature is and can be valued within political and economic decisions and in particular for going beyond the instrumental values that predominate in biodiversity policy debates and underpin the global biodiversity crisis. The report obviously could have gone further to consider non-anthropocentric values such as those related to animals’ sentience, and doing so would align better with other concerns such as Harmony with Nature and One Health. This omission of how people value sentient animals is therefore significant.

However, the report explicitly acknowledges that it may not have captured the full range of values linked to various knowledge systems, so it is hoped that future IPBES work can develop the report’s admirable approach further, to capture a fuller range of values including sentience-related values (and potentially others, on the logic that one omission may imply others). In the interim, since the IPBES’s assessment of the diverse values and valuation of nature is expected to contribute to achieving the 2050 Vision for Biodiversity, and the future post-2020 global biodiversity framework, there may need to be other routes to ensure animal sentience is adequately considered within these regimes for example at the Convention on Biological Diversity’s subsidiary bodies’ work.

The 2030 Agenda on sustainable development envisions “a world in which humanity lives in harmony with nature and in which wildlife and other living species are protected” [33], Art. 9. However, the considerations of health (SDG3), consumption and production, life below water (SDG14) and life on land (SDG15) do not benefit from any explicit and inherent targets relating to the health and wellbeing of the animals involved (other than a reference in Target 2.5 to their genetic diversity). However, the recent UN Global Sustainable Development Report (GSDR) has acknowledged animal welfare as a critical issue missing from the 2030 agenda and that strong governance should safeguard the wellbeing of both wildlife and domesticated animals [34].

The future consideration of animal welfare alongside other global concerns should help ensure that linked issues are not approached in silos, but in holistic and systems-based ways that consider issues from all major relevant lenses—including those of the animals themselves.

## 5. Conclusions

Animals’ sentience is an important part of nature and a significant reason to value nature in meaningful ways. These ways are partly non-anthropocentric insofar as sentience means that nature matters for nonhuman animals. They are partly anthropocentric insofar as sentience provides ways to evaluate nature and mobilise its protection, whether that is done for humans, animals or nature as a whole. Animal sentience is directly relevant to why nature has value, and also to why we recognise nature as having value. It is a core part of our environmental ethics and public concern worldwide. Recognising (or re-recognising) animal sentience, for animals in natural and anthropogenic environments, can help to inform and to motivate protection, collaboration and transformational action. The consideration of animal sentience should play a more significant and prominent role in UN considerations of nature, and specifically the value of nature, in alignment with transformative UN agendas.
